# Air Pollutants are associated with Dry Eye Disease in Urban Ophthalmic Outpatients: a Prevalence Study in China

**DOI:** 10.1186/s12967-019-1794-6

**Published:** 2019-02-15

**Authors:** Donghui Yu, Qinglong Deng, Jiwei Wang, Xing Chang, Shuxiao Wang, Renren Yang, Jinming Yu, Jing Yu

**Affiliations:** 1 Department of Ophthalmology, Shanghai Tenth People’s Hospital, School of Medicine, Tongji University, No. 301, Yanchang Road, Shanghai, China; 2 School of Medicine, Tongji University, No. 1239, Siping Road, Shanghai, China; 3 Institute of Clinical Epidemiology, School of Public Health and Key Laboratory of Public Health Safety, Ministry of Education, Fudan University, No. 138, Yixueyuan Road, Shanghai, China; 4 School of Environment, Tsinghua University, No. 1, Tsinghua Yuan, Haidian District, Beijing, China

**Keywords:** Air pollutant, China, Dry eye disease, Prevalence study

## Abstract

**Background:**

Although previous prevalence studies of DED were reported from some countries worldwide, national data are unavailable in China. We aimed to conduct an up-to-date national survey on the prevalence of DED in China and find out the potential risk factors including air pollutant.

**Methods:**

23,922 eligible outpatients were recruited from ophthalmic clinics of 32 cities in China in 2013 by registration orders. The patients’ demographic characteristics, history of keratorefractive surgery, diseases and medication history were collected and the daily air pollutant data in 2013. Multivariate logistic analysis was performed to identify the potential risk factors associated with DED. The association between related factors and dry eye diseases subtypes evaluated as p value and odds ratios (OR) with 95% confidence intervals (CI).

**Results:**

Among 23,922 outpatients, the prevalence of DED was 61.57%, and that of the male patients was 57.64% and of the female was 65.32% (P < 0.0001). Multivariate logistic regression suggested that the possible risk factors for DED included: female, older age, history of keratorefractive surgery, presence of arthritis, thyroid diseases, and antihistamine, diuretic, duodenal ulcer drugs, diazepam. Air pollutants including O_3_, PM_2.5_, and SO_2_ were also identified as the risk factors.

**Conclusion:**

The prevalence of DED among ophthalmic outpatients in China was considerably high. Age, gender, history of keratorefractive surgery, diseases, medication history, and air pollutants were associated with DED prevalence.

## Background

The concept of tear deficiency was first proposed in 1903 by Schirmer, who developed the Schirmer test 90 [1]. Several definitions about Dry Eye Disease (DED) were proposed in the next 100 years [2]. But the most widely accepted definition of Dry Eye was provided by the International Dry Eye Workshop in 2007, referring it as a multifactorial disease of the tears and ocular surface that results in symptoms of discomfort, visual disturbance, and tear film instability, with potential damage to the ocular surface. It is accompanied by increased osmolarity of the tear film and inflammation of the ocular surface [3]. In 2014, the Asia Dry Eye Society (ADES) published a new definition about DED as “Dry eye is a multifactorial disease characterized by unstable tear film causing a variety of symptoms and/or visual impairment, potentially accompanied by ocular surface damage.” [4].

Patients with DED complain most frequently of a scratchy or sandy (foreign body). Other common symptoms are itching, excessive mucus secretion, inability to produce tears, aburning sensation, photosensitivity, redness, pain, and difficulty in moving the lids. The most characteristic feature is interruption or absence of the tear meniscus at the lower lid margin. Early in the course of dry eye syndrome, vision is slightly impaired. As the condition worsens, discomfort can become disabling. In advanced cases, corneal ulceration, corneal thinning, and perforation may develop. Secondary bacterial ingestion occasionally occurs, and corneal scarring and vascularization may result in marked reduction in vision. Early treatment may prevent these complications.

Surveys showed wide range of DED prevalence, from around 2.1% to 35% and Asians was more susceptible than Caucasians and elderly women with are of higher risk [5, 6]. Some factors were independently and significantly associated with dry eye including history of arthritis, smoking status, caffeine use, history of thyroid disease, history of gout. Older age and illiteracy were also predictors of DED [7–9].

Air pollution has been linked to human diseases including respiratory disease, cardiovascular disease, kidney parenchyma cancer and so on [10–13]. Meanwhile, DED test outcomes assessing the ocular surface integrity and tear stability are climate dependent [14]. But there were few evidences about the relationship between DED and air pollution.

Despite several studies regarding the epidemiology and risk factors of DED, most of them were derived from populations with limited sample size, and few studies analyzed the risk factors comprehensively. In addition, a study focusing on Chinese DED population has not been reported and a study about the association between air pollution and DED is still lacking. In this setting, the current study aimed to understand the epidemiology of DED among ophthalmic outpatients from different cities in China, and to analyze the associated risk factors including air pollutants.

## Materials and methods

### Study population

In this study, 79 hospitals (or research centers) with ophthalmology clinics were randomly selected from 32 cities in China from July to December in 2013. Inclusion criterion of outpatients was a presence of at least one of these six symptoms: dry sensation, foreign body sensation, burning sensation, eyesight fatigue, discomfort, and vision fluctuation. Patients with other eye diseases such as conjunctivitis, glaucoma, and ocular trauma were excluded. Eventually, 23,922 eligible subjects were included in our study.

### Data collection

We collected the following information of the patients including: demographic characteristics (age and gender), history of keratorefractive surgery, history of diseases (diabetes, arthritis and thyroid diseases), and medication history (antihistamine, oral contraceptive, diuretic, duodenal ulcer drugs, and diazepam). Patients’ age were divided into 3 group based on the questionnaire. Clinical examinations including tear breakup time tests, Schirmer I tests and fluorescein staining were also conducted among these patients. All of the predictors were selected from clinic practice, expertise [15] and significant variables of Univariate analysis.

To study the relationship between air pollution and DED, we collected the daily air pollutant data (CO: average concentration in 24 h, NO_2_: average concentration in 24 h, O_3_: average concentration in 24 h, PM_10_: average concentration in 24 h, PM_2.5_: average concentration in 24 h, SO_2_: average concentration in 24 h) and yearly meteorological data (relative humidity, mean air pressure, and air temperature) of the 32 China cities from January 1, 2013 to December 31, 2013. Air pollutant data is kindly provided by School of Environment, Tsinghua University. Taking the 75th quantile of the pollutant concentration as “extreme value” (CO: 1.47 mg/m^3^, NO_2_: 52.83 μg/m^3^, O_3_: 79.06 μg/m^3^, PM_10_: 148.91 μg/m^3^, PM_2.5_: 81.21 μg/m^3^, SO_2_: 43.40 μg/m^3^), we calculated the number of days in which pollutant concentration exceeds “extreme value” in each city in 2013, and then we divided the number of days into two groups according to 75th percentile.

This DED survey was approved by Medical Research Ethics Committee of School of Public Health, Fudan University. Details and procedures of this study were indicated to all the patients by practitioners before the survey and clinical tests. Informed consent was obtained from all the subjects. Participants who disagreed with this survey would be excluded, thus, in this way we documented and insured all patient consents.

### Diagnosis of DED

Diagnosis was established according to a consensus of Chinese dry-eye diagnostic criteria from the Chinese Medical Association [16] as follows: (1) presence of at least one of the six symptoms: dry sensation, foreign body sensation, burning sensation, eyesight fatigue, discomfort and vision fluctuation; (2) TBUT ≤ 5 s or Schirmer I test (without anesthesia) ≤ 5 mm/5 min; (3) a positive diagnosis of fluorescein staining accompanied by one of the results: 5 s < TBUT ≤ 10 s or 5 mm/5 min < Schirmer I test (without anesthesia) ≤ 10 mm/5 min. The presence of (1) was essential for disease diagnosis. Subjects showing the presence of a combination of (1) and (2), or (1) and (3) were diagnosed with DED.

### Statistical analysis

Data analyses were performed using SAS 9.4 and R 3.4.1. The significance level was set at 0.05 and all the tests were two-sided. Descriptive statistics, and univariate analysis as well as multivariate analysis were both conducted. The DED prevalence of the whole population and different gender as well as age groups were calculated respectively. Pearson Chi square test was applied for enumeration data. A binary non-conditional logistic model was performed to conduct multivariate regression analysis. The dependent variable was the status of DED (1 = yes, 0 = no). The independent variables were the potential related factors as listed in Table 1, including demographic characteristics, history of keratorefractive surgery, diseases and medication history, as well as the daily air pollutant data. Yearly average of relative humidity, mean air pressure, and air temperature were controlled as confounding factor. The stepwise method was adopted to select significant independent variables.

**Table 1:** Characteristics and DED prevalence of the study population

Characteristics	Number of subjects (%)	Number of patients with DED	DED prevalence (%)	Chi square	P value
Gender				149.0810	< 0.0001
Male	11,686 (48.85)	6736	57.64		
Female	12,236 (51.15)	7993	65.32		
Age (years old)				351.0333	< 0.0001
< 25	4969 (20.77)	2579	51.90		
25–45	10,895 (45.54)	6648	61.02		
> 45	8058 (33.68)	5502	68.28		
Keratorefractive surgery				43.5570	< 0.0001
No	20,330 (84.98)	12,340	60.70		
Yes	3592 (15.02)	2389	66.51		
Diabetes				32.7384	< 0.0001
No	21,926 (91.66)	13,381	61.03		
Yes	1996 (8.34)	1348	67.54		
Arthritis				136.1486	< 0.0001
No	22,003 (91.98)	13,309	60.49		
Yes	1919 (8.02)	1420	74.00		
Thyroid diseases				11.1371	0.0008
No	22,503 (94.07)	13,796	61.31		
Yes	1419 (5.93)	933	65.75		
Antihistamine				65.9711	< 0.0001
No	22,414 (93.70)	13,652	60.91		
Yes	1508 (6.30)	1077	71.42		
OC				6.5310	0.0106
No	23,122 (96.66)	14,271	61.72		
Yes	800 (3.34)	458	57.25		
Diuretics				33.8723	< 0.0001
No	22,921 (95.82)	14,025	61.19		
Yes	1001 (4.18)	704	70.33		
DU drugs				57.8380	< 0.0001
No	23,361 (97.65)	14,297	61.20		
Yes	561 (2.35)	432	77.01		
Diazepam				72.7696	< 0.0001
No	23,170 (96.86)	14,154	61.09		
Yes	752 (3.14)	575	76.46		
CO (days)*				6.3735	0.0116
< 124	17,338 (72.48)	10,760	62.06		
≥ 124	6584 (27.52)	3969	60.28		
NO_2_ (days)*				2.9279	0.0871
< 150	15,968 (66.75)	9771	61.19		
≥ 150	7954 (33.25)	4958	62.33		
O_3_ (days)*				1349.9078	< 0.0001
< 125	17,770 (74.28)	9733	54.77		
≥ 125	6152 (25.72)	4996	81.21		
PM_10_ (days)*				63.8984	< 0.0001
< 102	15,071 (63.00)	8989	59.64		
≥ 102	8851 (37.00)	5740	64.85		
PM_2.5_ (days)*				5.0322	0.0249
< 143	17,195 (71.88)	10,663	62.01		
≥ 143	6727 (28.12)	4066	60.44		
SO_2_ (days)*				256.7553	< 0.0001
< 101	17,555 (73.38)	10,276	58.54		
≥ 101	6367 (26.62)	4453	69.94		
Total	23,922 (100.00)	14,729	61.57	–	–

* The number of days in which the pollutant concentration exceeds the “extreme value”

## Results

### General information

Among 23,922 outpatients, 51.16% were female, and 45.54% were aged from 25 to 45 years old. There were few patients with the history of keratorefractive surgery (15.02%), diabetes (8.34%), arthritis (8.02%), or thyroid diseases (5.93%). Also, few of them had the history of using antihistamine, oral contraceptive (OC), diuretic, duodenal ulcer drugs (DU drugs) and diazepam. The corresponding percentage were 6.3%, 3.34%, 4.18%, 2.35%, and 3.14%, respectively.

Detailed information and the DED prevalence among different groups were as shown in Table 1. Significant differences of DED prevalence could be found in different gender (P < 0.0001); age groups (P < 0.0001); history of keratorefractive surgery (P < 0.0001), diabetes (P < 0.0001), arthritis (P < 0.0001), and thyroid diseases (P = 0.0008); history of using antihistamine (P < 0.0001), OC (P = 0.0106), diuretic (P < 0.0001), DU drugs (P < 0.0001), and diazepam (P < 0.0001). There were also significant differences of DED prevalence among the following air pollutants: CO (P = 0.0116), O_3_ (P < 0.0001), PM_10_ (P < 0.0001), PM_2.5_ (P = 0.0249) and SO_2_ (P < 0.0001).

The characteristics of the air pollutants in different cities was shown in Table 2 (only the first and last three cities sorted by the days that the pollutant concentration exceeds the “extreme value” were listed).

**Table 2:** Characteristics of the air pollutants in different cities

Type	City	Median (P25–P75)	Days*
CO	Tianjin	1.72 (1.37–2.29)	250
	Xi’an	1.70 (1.26–2.47)	211
	Xingtai	1.54 (1.11–2.46)	198
	–	–	–
	Guangzhou	1.00 (0.89–1.19)	27
	Fuzhou	0.73 (0.63–0.94)	10
	Guiyang	0.77 (0.63–0.95)	10
NO_2_	Xingtai	61.79 (47.67–82.99)	232
	Chengdu	51.76 (42.45–65.44)	178
	Ji’nan	49.70 (35.48–68.42)	162
	–	–	–
	Kunming	36.50 (28.90–44.51)	26
	Dalian	28.94 (23.58–37.33)	17
	Guiyang	31.05 (24.03–39.59)	16
O_3_	Wuhan	79.06 (51.67–110.33)	181
	Shanghai	77.55 (58.89–96.12)	165
	Ji’nan	72.48 (40.46–102.40)	160
	–	–	–
	Zunyi	64.79 (57.48–73.13)	31
	Hefei	55.23 (38.77–67.33)	29
	Fuzhou	49.42 (39.63–62.93)	27
PM_10_	Xingtai	251.43 (172.92–359.36)	308
	Ji’nan	166.03 (120.06–237.78)	215
	Xi’an	143.05 (98.79–218.76)	173
	–	–	–
	Shenzhen	59.83 (38.44–94.45)	19
	Kunming	72.19 (46.77–99.89)	15
	Fuzhou	62.29 (45.56–87.95)	14
PM_2.5_	Xingtai	119.29 (78.09–196.94)	258
	Ji’nan	84.93 (59.76–129.42)	192
	Tianjin	80.58 (54.03–113.46)	178
	–	–	–
	Shenzhen	36.92 (21.18–58.11)	27
	Fuzhou	31.65 (20.53–49.52)	12
	Kunming	37.88 (24.00–53.04)	9
SO_2_	Xingtai	84.58 (51.71–137.72)	291
	Ji’nan	61.47 (40.98–104.46)	256
	Shenyang	40.98 (24.26–98.12)	169
	–	–	–
	Wenzhou	20.14 (13.46–29.00)	13
	Guangzhou	20.19 (15.07–26.22)	6
	Shenzhen	9.83 (7.43–14.57)	1

* The days of a specific city that the pollutant concentration exceeds the “extreme value”

### Epidemiological characteristics of DED

**Fig. 1:**
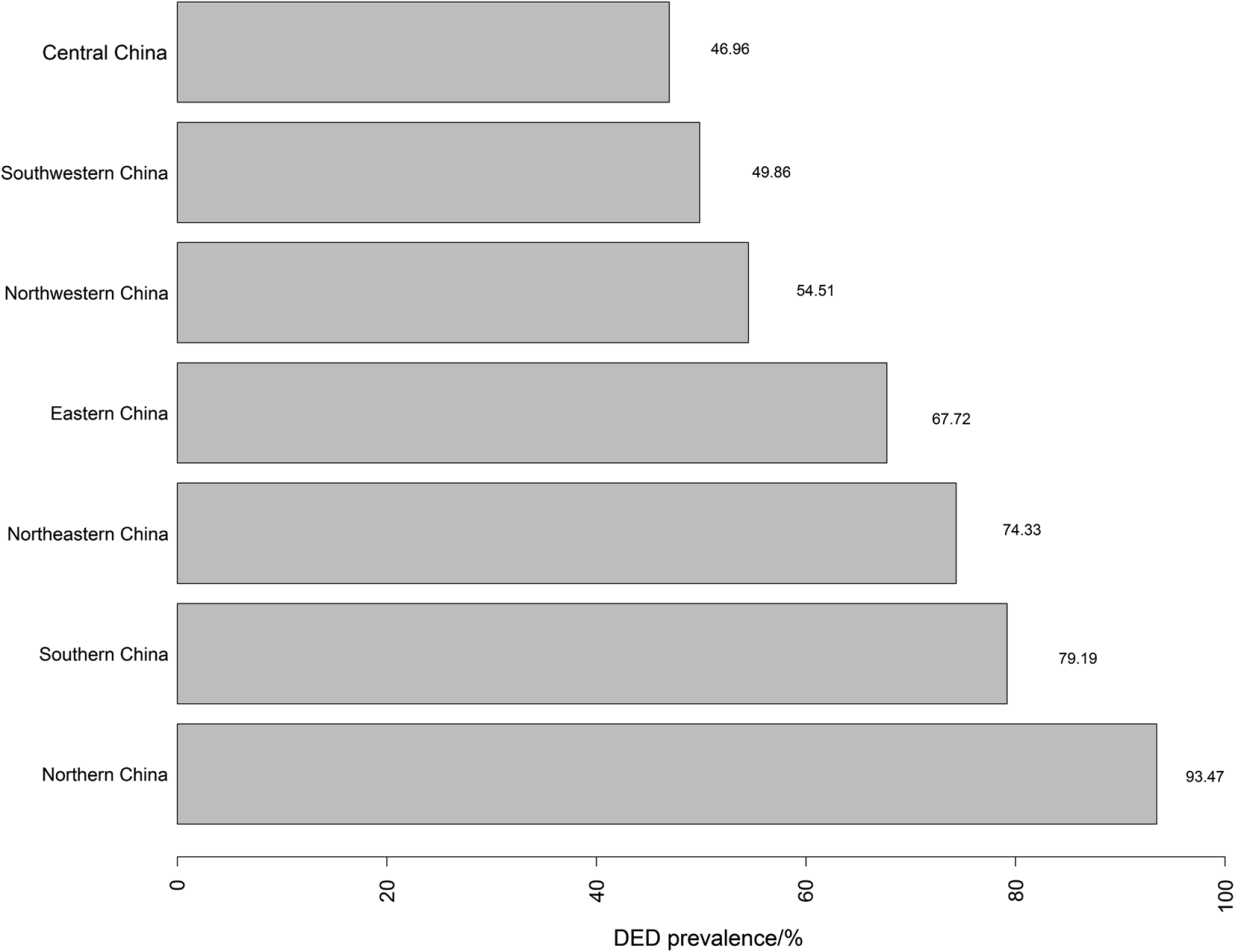
The prevalence of DED in different areas

**Fig. 2:**
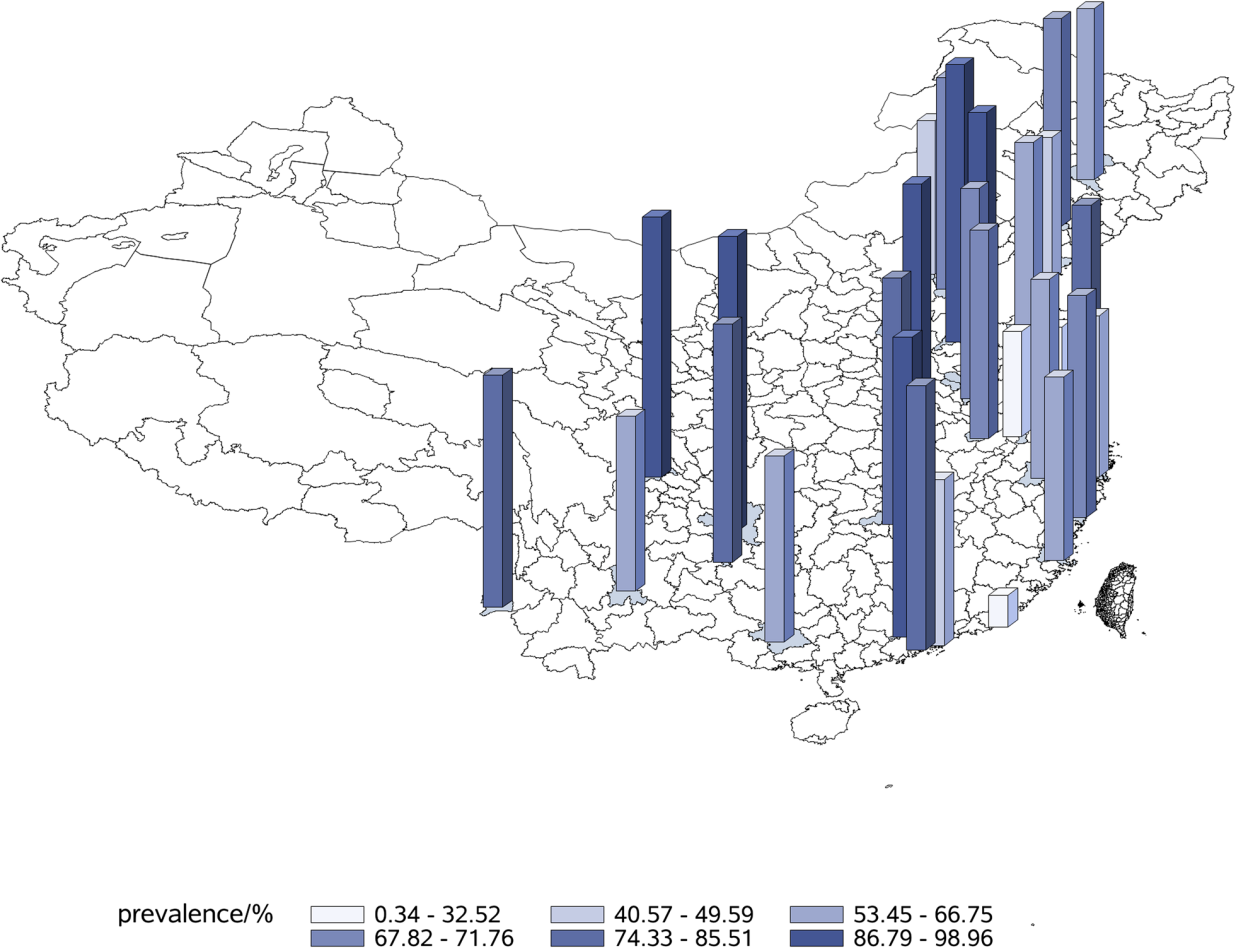
The prevalence of DED in different cities

The prevalence of DED was 61.57% among 23,922 outpatients, and that of the male patients was 57.64% and of the female patients was 65.32% (P < 0.0001). The patients whose age was younger than 25 years old had a prevalence of 51.90%, patients whose age was between 25 and 45 years old had a prevalence of 61.02%, and patients whose age was older than 45 years old had a prevalence of 68.28% (P < 0.0001). The prevalence of DED in different areas and cities were shown in Figs. 1 and 2. The highest prevalence of DED in China was in northern area and the lowest was in central area. The cities with the highest prevalence and lowest prevalence of DED were Zunyi and Shantou, respectively.

### Multivariate logistic regression analysis of risk factors for DED

Multivariate logistic regression analysis suggested that the potential risk factors for DED were: female, older age, history of keratorefractive surgery, presence of arthritis, thyroid diseases, history of using antihistamine, diuretic, DU drugs, diazepam, and air pollutants including O_3_, PM_2.5_ and SO_2_. While, history of using OC and the air pollutant CO and NO_2_ may be the potential protective factors for DED. However, history of diabetes and PM_10_ were not associated with DED. Detailed information was shown in Table 3.

**Table 3:** Multivariate logistic regression analysis of risk factors for DED

Characteristics	β	OR	95% CI	Wald χ^2^	P value
Gender					
Male	Ref	Ref	–	–	–
Female	0.4079	1.504	(1.421, 1.592)	197.6858	< 0.0001
Age (years old)					
< 25	Ref	Ref	–	–	–
25–45	0.4540	1.575	(1.462, 1.696)	143.6879	< 0.0001
> 45	0.9010	2.462	(2.267, 2.675)	453.8747	< 0.0001
Keratorefractive surgery					
No	Ref	Ref	–	–	–
Yes	0.5449	1.724	(1.588, 1.874)	166.8826	< 0.0001
Arthritis					
No	Ref	Ref	–	–	–
Yes	0.5060	1.659	(1.483, 1.857)	77.8043	< 0.0001
Thyroid diseases					
No	Ref	Ref	–	–	–
Yes	0.2502	1.284	(1.139, 1.449)	16.6104	< 0.0001
Antihistamine					
No	Ref	Ref	–	–	–
Yes	0.4004	1.492	(1.321, 1.689)	40.9029	< 0.0001
OC					
No	Ref	Ref	–	–	–
Yes	− 0.3023	0.739	(0.632, 0.865)	14.3181	0.0002
Diuretics					
No	Ref	Ref	–	–	–
Yes	0.2087	1.232	(1.061, 1.433)	7.4087	0.0065
DU drugs					
No	Ref	Ref	–	–	–
Yes	0.5263	1.693	(1.372, 2.101)	23.4873	< 0.0001
Diazepam					
No	Ref	Ref	–	–	–
Yes	0.4334	1.542	(1.289, 1.855)	21.7865	< 0.0001
CO (days)*					
< 124	Ref	Ref	–	–	–
≥ 124	− 0.7163	0.489	(0.424, 0.563)	97.1635	< 0.0001
NO_2_ (days)*					
< 150	Ref	Ref	–	–	–
≥ 150	− 0.1010	0904	(0.834, 0.980)	5.9988	0.0143
O_3_ (days)*					
< 125	Ref	Ref	–	–	–
≥ 125	1.3777	3.966	(3.666, 4.293)	1171.6734	< 0.0001
PM_2.5_ (days)*					
< 143	Ref	Ref	–	–	–
≥ 143	0.6971	2.008	(1.789, 2.255)	139.8619	< 0.0001
SO_2_ (days)*					
< 101	Ref	Ref	–	–	–
≥ 101	0.4941	1.639	(1.503, 1.788)	123.9515	< 0.0001

Factors controlled: relative humidity, mean air pressure, and air temperature

* The number of days in which the pollutant concentration exceeds “extreme value”

## Discussion

DED were very common in this population-based sample of ophthalmic outpatients in China. This study suggested that the prevalence of DED in outpatients increased with age and female had a higher prevalence. In previous reports, the prevalence of dry eye disease varied according to the diagnostic criteria, population, and age distribution. But this study of ophthalmic outpatients in China indicated a higher prevalence of dry eye compared with other studies [5, 6] because we got the data from ophthalmic clinics instead of communities.

In recent years, more and more attention has been paid to the relationship between dry eye disease and environmental factors. As a type of dry eye disease, dry eye caused by environmental factors may be affected by various aspects [17] including high altitude, wind, and air pollution [18]. But there was no consistent view about the effect of different pollutants on DED. There was a study [19] about the relationship between outdoor air pollution and DED: higher O_3_ levels and lower humidity levels were associated with higher DED prevalence in the Korean population, while PM10 level was not associated with DED. However, in the same country, another study [20] showed a different conclusion: lower humidity and longer sunshine duration were significantly associated with higher DED prevalence and among air pollutants, SO_2_ was associated with DED, while NO_2_, O_3_, CO, and PM_10_ were not. The phosphorylation and interaction of FAK/paxillin, RhoA activity as well as actin reorganization were suppressed by PM_2.5_ exposure. Moreover, formation of ROS might play a role in the action of PM_2.5_ [21]. PM_2.5_ induced apoptosis. Corneal epithelial cells were damaged and PM_2.5_ exposure could result in delay of corneal epithelium wound healing by inhibiting cell migration [22, 23]. It may be one of the pathogenic factors of DED caused by PM_2.5_. DED is associated with the inflammatory cascade of Mitogen-Activated Protein (MAP) kinase and Nuclear Factor κB (NF-κB) signaling pathway. Meanwhile, various proinflammatory cytokines (e.g. IL-1α, IL-1β, TNF-α) and Matrix metalloproteinase (e.g. MMP-1, MMP-3, MMP-9, MMP-13) were included in [24–27]. The O_3_-induced expression of proinflammatory cytokines requires the activation of the epidermal growth factor receptor/MEK/ERK and MKK4/p38 mitogen-activated signaling pathways and NF-κB signaling pathways [28, 29]. That is probably the pathogeny for DED through O_3_. In this study, air pollutants including O_3_, PM_2.5_ and SO_2_ were the potential risk factors for DED and PM10 and NO_2_ were not associated with DED. However, CO may be the potential protective factor for DED. A study in America showed that increased ambient NO_2_ was consistently associated with increased prevalence of allergic sensitization [30]. But no evidences indicated the relationship between NO_2_ and DED prevalence. These findings need to be confirmed by large prospective studies and the mechanism remains to be discovered. As a ecological study, some problems still exist. Although some confounding factors were controlled in this study including relative humidity, mean air pressure, and air temperature, more confounding factors cannot be controlled by our study (such as individuals’ life styles and gene). It is the defect of our study and other ecological studies.

Many diseases were associated with DED. Our study showed that the history of arthritis diseases was one of the risk factors of DED. Dry eye is common in early Graves’ ophthalmopathy even in the absence of apparent exophthalmos and reduced corneal sensitivity [31]. In thyroid diseases, autoantibodies may bind to lacrimal TSH receptor and, perhaps via aberrant signal transduction, contribute to lacrimal gland impairment and, hence, dry eye syndrome [32]. A study showed that the tear film and cornea were damaged in newly non-exophthalmic Graves’ disease subjects [33]. Meanwhile, another survey [34] showed the significantly higher tear film osmolarity in patients with thyroid ophthalmopathy was most likely due to the increased proptosis and lid fissure width. These conditions may lead to injury of the ocular surface. Tear film instability and tear hyperosmolarity played major roles in the vicious circle of DED pathology. Hyperosmolarity directly caused cell damage and nerve stimulation and triggered inflammatory cascades. These cascades then contributed to further cell damage, including loss of mucin-producing goblet cells [35]. This study showed the same result as these previous studies. In the past decades of years, many researches have studied the mechanism of diabetes associated with dry eye in the structure of tear film [36], tear secretion and its disfunction of the autonomic nervous system in diabetic patients [37]. A recent study showed that corneal sensation was reduced in diabetic patients and progresses with the severity of neuropathy, suggesting that corneal nerve fibre damaged accompanying somatic nerve fibre damage [38]. These findings were consistent with a meta-analysis in mainland China: diabetes is a potential risk factor for DED [39]. However, data in this research indicated that diabetes was not significantly associated with DED. One explanation is that some of these outpatients have had DED before they were diagnosed with diabetes. This is one of the limitations of cross-sectional study. One study [40] revealed that meibomian gland function is influenced after ocular surgery accompanying structural changes and these were correlated with increased ocular symptom scores. Therefore, it could elucidate the development of dry eye related to ocular surgery. Our study also demonstrated an association between corneal refractive surgery and DED, which is consistent with the findings of previous studies.

Many different drugs will influence on DED. The effect of antihistamines on dry eye has been unanimously recognized by scholars all over the world and this study got the same conclusion. The finding in Wisconsin [41] showed that dry eye could be related to the use of antihistamines that have anticholinergic properties owing to high muscarinic receptor binding. Systemic antihistamines, including old and newer generations, have been shown to potentially cause clinically meaningful damage to the ocular surface as a result of anti-muscarinic activity on the M3 receptors that results in a decrease in both aqueous and mucin tear [42]. There is no consensus of opinion about the effect of estrogen on DED. A previous study [43] showed phytoestrogen supplementation can significantly improve the signs and symptoms of dry eye syndrome in postmenopausal women. But latest survey showed another opinion: Serum hormone levels did not contribute significantly to dry eye symptoms. In our study, OC, a kind of estrogen, showed significant protective effect on DED. Whether estrogen can affect DED and what’s the potential mechanism needs further studies. As for diuretics, this study showed that diuretics was a risk factor for DED. However, there was no relevant research supporting this finding and a hypothesis is that it might be related to the function of diuretics that can reduce systemic circulation volume. But this assumption needs to be verified by experiment in future. Previous study indicated that medications prescribed for psychiatric conditions may precipitate DED [44]. It may be the reason that diazepam is a risk factor of DED. Meanwhile, the results of several previous researches suggested a reciprocal relationship between DED and sleep disorders, particularly in elderly patients [45, 46]. Diazepam is generally used by patients with sleep disorders. The relationship between diazepam and DED discovered in this study may be caused by sleep disorders instead of the effect of diazepam, and this remains further exploration.

This cross-sectional study revealed the prevalence and risk factors for DED among ophthalmic outpatients in China. However, exposure to sunlight was not considerated and factors affecting the severity of DED were not clear. Consequently, a further study about the potential influence factors for sunlight and the severity of DED was expected in future. Meanwhile, Besides, the findings of this research should be generalized to a population derived from communities rather than ophthalmic clinics. Thirdly, a prospective cohort study was expected to demonstrate the risk factors of DED. Last but not least, studies in other countries, regions, or ethnic groups were needed with equal importance.

## Conclusion

This present study suggested that the prevalence of DED among ophthalmic outpatients in China was considerably high. The significant associated factors for DED included age, gender, history of keratorefractive surgery, diseases (arthritis, thyroid diseases), medication history (using antihistamine, diuretic, duodenal ulcer drugs, diazepam, oral contraceptive), and air pollutants (O_3_, PM_2.5_, SO_2_, CO, NO_2_).

## Abbreviations

DED: dry eye disease
OR: odds ratios
CI: confidence intervals
P: P value
O_3_: ozone
NO_2_: nitrogen dioxide
PM_2.5_: particulate matter 2.5
PM_10_: particulate matter 10
SO_2_: sulfur dioxide
OC: oral contraceptive
